# An Immunochromatographic Test Strip for Rapid Quantitative Control of Monoclonal Antibodies against Programmed Cell Death Protein 1

**DOI:** 10.3390/molecules29133046

**Published:** 2024-06-27

**Authors:** Jingyi Zhang, Congmei Lin, Feng Li, Xinhao Wei, Yusen Chen, Yanyong Fu, Xiaoping Yu, Biao Zhang, Zihong Ye

**Affiliations:** 1 Zhejiang Provincial Key Laboratory of Biometrology and Inspection & Quarantine, College of Life Sciences, China Jiliang University, Xueyuan Street, Xiasha Higher Education District, Hangzhou 310018, China; zhangjingyi@cjlu.edu.cn (J.Z.); lincongmei@cjlu.edu.cn (C.L.); lifeng@cjlu.edu.cn (F.L.); winxinhao@cjlu.edu.cn (X.W.); chenyusen@cjlu.edu.cn (Y.C.); yxp@cjlu.edu.cn (X.Y.); 2 Hangzhou Mingzhi Medical Laboratory Co., Ltd., Hangzhou 310020, China; fuyanyong@163.com

**Keywords:** PD-1 monoclonal antibody, immunochromatographic test strip, AuNPs enlargement method, test strip reader

## Abstract

Cancer is one of the major public health challenges in the world, which is characterized by rapid progression and high mortality. Immunotherapy, represented by PD-1 monoclonal antibody, has significantly improved the efficacy of malignant tumors and has become one of the most popular immunotherapy methods at present. Therefore, there is an increasing demand for novel detection methods for PD-1 monoclonal antibodies. The aim of this work was to establish a rapid, simple, and sensitive immunochromatographic test strip (ICTS) based on the AuNPs enlargement for both visual and instrumental detection of the PD-1 monoclonal antibody concentration. The mixed solution of NH_2_OH·HCl and HAuCl_4_ was used as an enhancement solution to lower the detection limit and achieve higher sensitivity. A test strip reader was used to construct a visualized quantitative detection standard curve for the PD-1 monoclonal antibody concentration. The LOD was 1.58 ng/mL through a triple signal-to-noise ratio. The detection time was within 10 min. The constructed test strips can rapidly, accurately, and efficiently detect the concentration of PD-1 monoclonal antibody in real samples.

## 1. Introduction

In recent years, the global market of biological products has grown rapidly. The market size of protein-based biological products accounts for nearly 90% and maintains an annual growth rate of nearly 10%, mainly including antibodies such as PD-1 monoclonal antibody, cytokines such as interleukin, enzymes such as thrombin, and antibody–drug conjugates, which are mostly developed by European and American countries. Recently, China’s production capacity of protein biological products has developed, but the overall level still lags behind Europe and the United States, especially the low level of quality control, resulting in insufficient market competitiveness of domestic products. Therefore, improving the product quality detection ability is an important research field of protein biological products.

Programmed cell death protein 1 (PD-1), a member of the CD28/B7 family, is a checkpoint co-suppressor expressed on the surface of immune cells, playing a crucial role in the negative regulation of the immune response [1,2]. Programmed death ligand 1 (PD-L1), the main ligand of PD-1, exerts its function of inhibiting T cell activity by binding to PD-1, preventing T cells from recognizing cancer cells [3]. Studies have shown that anti-tumor immunity can be enhanced by blocking the PD-1/PD-L1 pathway [4,5]. Immunotherapy, represented by the PD-1 monoclonal antibody, has dramatically improved the efficacy of malignant tumors and has become one of the most popular immunotherapy methods at present [6]. PD-1 antibody drugs include Pabolizumab, Sindilizumab injection, Tirellizumab injection, etc., which are used in the treatment of Hodgkin’s lymphoma, lung cancer, melanoma, colorectal cancer, primary liver cancer, etc. [7,8,9,10,11,12,13,14,15]. In the in-depth clinical research on the PD-1 monoclonal antibody, the quality detection of the antibody is so important to ensure that the antibody has a high purity and titer. Therefore, there is an increasing demand for new PD-1 monoclonal antibody detection methods. Puszkiel et al. reported an ELISA method for the quantification of nivolumab in plasma from NSCLC patients [16]. The detection limit of this method was 5 g/mL. Iwamoto et al. validated the LC–MS/MS analysis of immune checkpoint inhibitor nivolumab in human plasma using a Fab peptide-selective quantitation method; the detection limit was 0.977 g/mL [17]. Pluim et al. used an enzyme-linked immunosorbent assay (ELISA) for the quantification of nivolumab and pembrolizumab in human serum and cerebrospinal fluid with a detection limit of 2 ng/mL [18]. So far, commonly used detection methods for the PD-1 antibody concentration have the benefits of excellent detectability and high specificity, but the drawbacks include a high detection cost, complex operation, and dependence on large instruments. As mentioned above, instrument methods, such as LC/MS/MS, involve sample processing, such as denaturation, reduction, alkylation, and digestion of protein analytes, which require a long experiment time and rely on large instruments. An immunoassay, such as the ELISA method, still requires approximately 2 h of time and a microplate reader [19]. Consequently, it is crucial to develop a more rapid, simple, and sensitive method for detecting the PD-1 antibody concentration. Immunochromatographic test strips (ICTS) have been considered to be attractive sensing tools because of their rapidity, portability, low cost, and on-site detection format. This method does not need sophisticated instruments, and the operation is very easy. The detection time is as little as 5–15 min [20,21]. ICTS have been extensively used in food hazard detection, environmental monitoring, in vitro diagnosis, medical diagnosis, and other aspects [22,23].

**Scheme 1:**
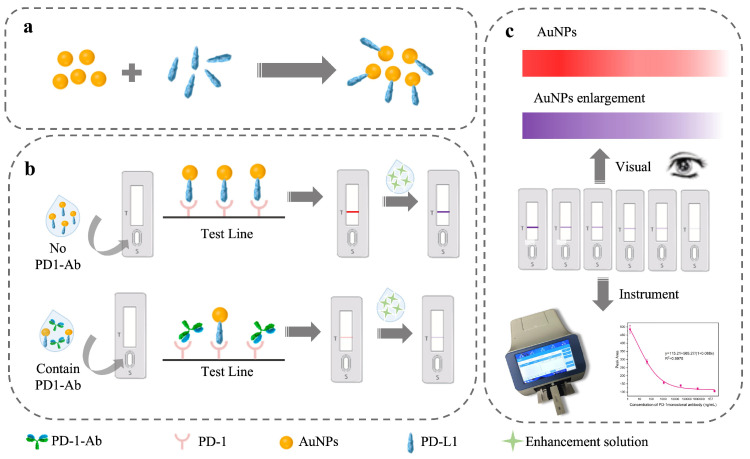
Schematic illustration of immunochromatographic strips. (**a**) Preparation of AuNPs-PD-L1; (**b**) Detection principle; (**c**) Result reading.

The aim of this work was to establish a rapid, simple, and sensitive immunochromatographic test strip (ICTS) for both visual and instrumental detection of the concentration of the PD-1 monoclonal antibody. The commonly used method for antibody detection is non-competitive immunochromatography, but one kind of additional antibody needs to be added. For the purpose of cost reduction, in this work, competitive immunochromatography was used, which would not be too expensive. The principle of this work was shown in Scheme 1. AuNPs-PD-L1 (a compound of PD-L1 and AuNPs) is used as the probe. PD-1 is fixed to the T-line of the test strip. When the concentration of PD-1 monoclonal antibody is 0 mg/mL, AuNPs-PD-L1 will bind to PD-1, and the color of the T-line is the darkest at this time. When the concentration of the PD-1 monoclonal antibody increases, AuNPs-PD-L1 and the PD-1 monoclonal antibody will compete to bind PD-1, and the color of the T-line gradually becomes lighter. Qualitative detection of PD-1 monoclonal antibody is performed using the naked eye, while quantification is performed using a test strip reader. In order to achieve the optimal test performance, all assay parameters were investigated [24].

## 2. Results and Discussion

### 2.1. Characterization and Identification of AuNPs and AuNPs-PD-L1

**Figure 1:**
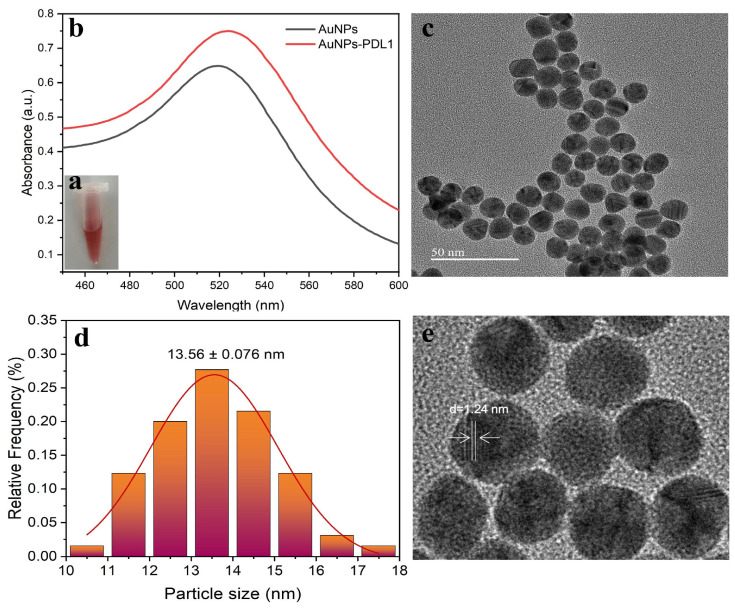
Characterization analysis of AuNPs and AuNPs-PD-L1. (**a**) An image of AuNPs to the naked eye; (**b**) Visible absorption spectra of AuNPs and AuNPs-PD-L1; (**c**) TEM image of AuNPs; (**d**) The diameter analysis of AuNPs; (**e**) The lattice spacing of AuNPs.

The color of AuNPs depended on their diameter, and the AuNPs with the diameter of 10–20 nm were wine red [25]. As shown in Figure 1a, the prepared AuNPs were wine red to the naked eye, with a clear color and no impurities. Furthermore, the prepared AuNPs and AuNPs-PD-L1 were scanned by a spectrometer. As shown in Figure 1b, the prepared AuNPs had an obvious single characteristic absorption peak at 519 nm. When combined with PD-L1, the peak value shifted from 519 nm to 524 nm, indicating that AuNPs and PD-L1 were combined successfully. Moreover, as shown by TEM characterization in Figure 1c, AuNPs had a uniform diameter and good dispersibility, and there was no agglomeration phenomenon. According to the diameter analysis in Figure 1d, the diameter of the prepared AuNPs was 13.56 nm, and the lattice spacing was 1.24 nm, as shown in Figure 1e. The results showed that the AuNPs were prepared successfully in the small and uniform diameter.

### 2.2. Optimization Results of Experimental Conditions

As shown in Figure S1a, the PH of AuNPs was adjusted with K_2_CO_3_. When 1 mL of AuNPs was added with 2 μL of K_2_CO_3_, the color of AuNPs was wine red and would not change any more. The results showed that the optimal PH was 1 mL of AuNPs added with 2 μL of 0.2 mol/L potassium carbonate. As shown in Figure S1b, when 1 mL of AuNPs was added with 1 μL of PD-L1, the color of AuNPs changed from blue to red, indicating that the dosage of 1 μL of PD-L1 to 1 mL of AuNPs was the most appropriate.

**Figure 2:**
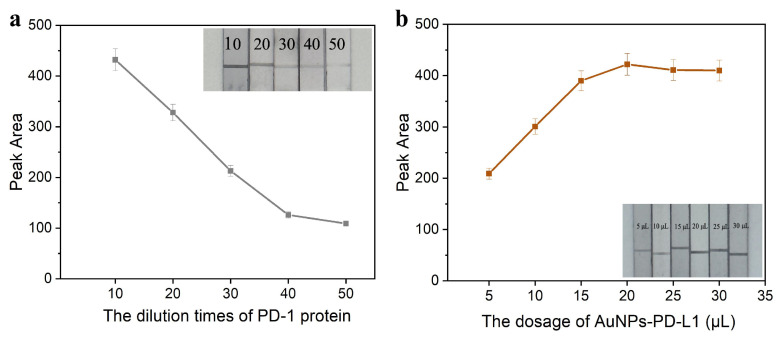
Optimization results of experimental conditions. (**a**) Optimization of PD-1 protein dilution times (the concentration of PD-1 protein was 0.166 mg/mL, 0.083 mg/mL, 0.055 mg/mL, 0.042 mg/mL, and 0.033 mg/mL, n = 3). (**b**) Optimization of AuNPs-PD-L1 dosage (n = 3).

As shown in Figure 2a, when PD-1 protein was diluted 10 times (the concentration of PD-1 protein was 0.17 mg/mL), the T-line color of the test strip was the darkest, and the peak area was 432. When increasing the dilution times of PD-1 protein, the color of the T-line gradually became lighter, and the peak area declined. Therefore, a dilution of 10 times was the best dilution of PD-1 protein. As shown in Figure 2b, when increasing the dosage of AuNPs-PD-L1, the color of the T-line gradually became darker. When the dosage of AuNPs-PD-L1 was 20 μL, the color of the T-line was obvious, and the peak area was 422. Subsequently, when increasing the dosage of AuNPs-PD-L1, the color of the T-line, as well as the peak area, were basically unchanged. Therefore, the optimal dosage of AuNPs-PD-L1 was 20 μL.

As shown in Figure S1c, when ultra-pure water was used, the T-line had no color. When PB buffer was used, the color of the T-line was the darkest, but the test result was a false-positive. When PBST and PBS buffer were used, the T-line color was moderate, and when PBS buffer was used, the T-line was darker and clearer. Therefore, the best loading buffer was the PBS buffer. As shown in Figure S1d, when the ratio of HAuCl_4_ to NH_2_OH·HCl was 1:5, the color of the T-line appeared darker, but when different concentrations of PD-1 monoclonal antibody were added, the T-line color did not change, indicating the presence of false-positive results. When the ratio was 1:15, the color of the T-line was lighter, indicating a decrease in sensitivity. When the ratio was 1:10, distinct colors were visible on the T-line, with no false-positive results. Therefore, the optimal ratio between HAuCl_4_ and NH_2_OH·HCl was chosen as 1:10.

### 2.3. Results of Analytical Performance

**Figure 3:**
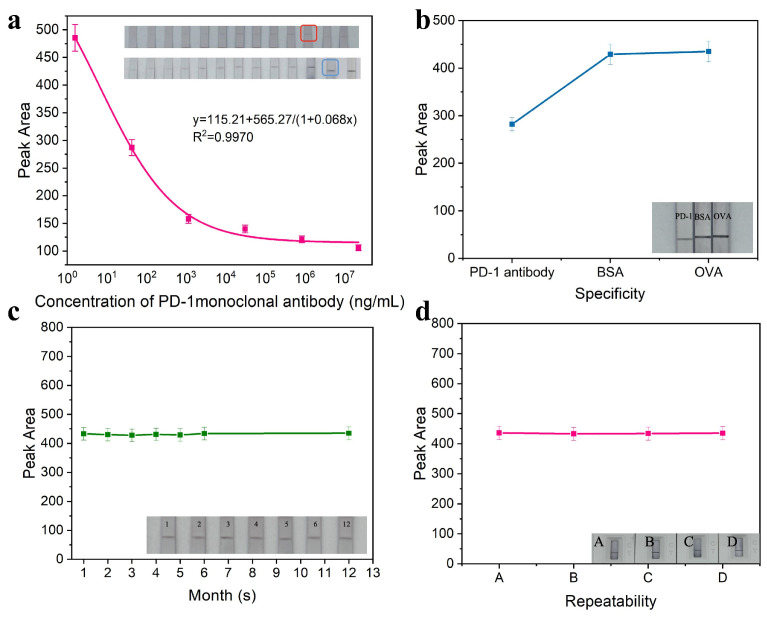
Performance identification of immunochromatographic test strips. (**a**) Detection limit (the PD-1 antibody concentrations from left to right were 0.28 mg/mL, 0.09 mg/mL, 0.03 mg/mL, 0.01 mg/mL, 3.4 mg/mL, 1.12 μg/mL, 0.38 μg/mL, 0.13 μg/mL, 0.04 μg/mL, 14.0 ng/mL, 4.7 ng/mL, 1.58 ng/mL, and 0 ng/mL); (**b**) Specificity (the concentrations of PD-1 antibody, BSA, and OVA were 0.5 mg/mL, n = 3); (**c**) Stability (n = 3); (**d**) Repeatability (n = 3).

As shown in Figure 3a, when the concentration of the PD-1 monoclonal antibody decreased, the T-line color of the test strip gradually became darker. When the concentration of the PD-1 monoclonal antibody was 1.58 ng/mL, the peak area was 485. With the decrease in the concentration of the PD-1 monoclonal antibody, the peak area did not change. Therefore, the LOD was 1.58 ng/mL through a triple signal-to-noise ratio. As shown in Figure 3b, the T-line color of the test strip with the addition of the PD-1 monoclonal antibody became lighter, and the peak area was about 435. There was no significant change in the T-line color of the test strip with adding BSA and OVA, with peak areas of 429 and 435, respectively. This indicated that the test strip did not react with other proteins and had high specificity. As shown in Figure 3c, the test strips were tested at different times, the test results were basically consistent, and the peak area was about 435. The strips were clear and stable. As shown in Figure 3d, the thickness and uniformity of the T-line of the test strips in batches A, B, C, and D were basically consistent, and the peak area was about 432, indicating that the test strip had good repeatability.

As shown in Table 1, the recovery rates of samples in PBS buffer and human serum were 95.00–107.40% and 96.67–99.28%, respectively. The relative standard deviations were 5.06–8.81% and 3.10–9.29%, respectively.

As shown in Table S2, the recovery rates of samples in the PBS buffer and human serum via the ELISA method were 99.33–101.15% and 98.33–101.33%, respectively. The relative standard deviations were 2.09–9.84% and 1.37–9.50%, which were consistent with the results of the immunochromatographic test strip, indicating the test strip has good accuracy and precision and can be applied in detecting human serum.

As shown in Table 2, two real samples were detected; the measurement value of the ICTS method was 4.82 mg/mL and 9.82 mg/mL, respectively, close to the actual concentration of the real samples. The relative standard deviation of the ICTS method was 1.43–2.04%, while that of the ELISA method was 1.49–1.87%, indicating that the immunochromatographic test strip was applicable in real samples and had practical application value.

## 3. Materials and Methods

### 3.1. Materials and Reagents

Human Anti-PDCD1/PD-1/CD279 Reference Antibody (pembrolizumab), Human PD-1 protein, and PD-L1 protein were purchased from Sanyou Biopharmaceutical Co., LTD. (Shanghai, China). Tetrachloro-auric acid (HAuCl_4_) and OVA were purchased from Sigma-Aldrich (Saint Louis, MO, USA). Hydroxylamine hydrochloride (NH_2_OH·HCl) and trisodium citrate were purchased from Shanghai Macklin Biochemical Technology Co., LTD. (Shanghai, China). Bovine Serum Albumin (BSA) was purchased from Beijing Solarbio Science & Technology Co., Ltd. (Beijing, China). PBST, PBS, PB, and Tween-20 were purchased from Aladdin Reagent (Shanghai) Co., LTD. (Shanghai, China). All elements of immunochromatographic test strip (Sample pad, Nitrocellulose membrane (Catalogue No. NCKB15A-25, Model No. NCKB15A, pore size: 15 um, Batch No. SRB022022, Width: 25 mm), absorbent pad and PVC plywood) were obtained from the Shanghai Gold Label Biological Company (Shanghai, China).

### 3.2. Preparation of AuNPs-PD-L1

According to previous work [26], AuNPs were prepared as follows: a mixture of 100 μL 10% (*v*/*v*) HAuCl_4_ and 99.9 mL of ultra-pure water was boiled. Then, 2.5 mL 1% citric acid was added quickly and heated for 15 min. When the color of the solution gradually turned wine red, heating was stopped immediately. The solution was cooled to room temperature. Finally, the solution was kept away from light and stored in a refrigerator at 4 °C. The prepared AuNPs were characterized by a spectrometer and Transmission Electron Microscope.

AuNPs-PD-L1 was prepared as follows: PD-L1 was added to 1 mL AuNPs, and the PH was adjusted with 0.2 M K_2_CO_3_. After mixing well, the PD-L1 was placed in a refrigerator at 4 °C for 1 h. Then, 20 μL of 20% BSA was added, and the mixed solution was once again placed at 4 °C for 1 h. The reaction solution was centrifuged at high speed (11,180× *g*) for 15 min. The precipitation was dispersed in 100 μL of buffer solution (0.02 mol/L PB, pH of 7.4) to obtain the AuNPs-PD-L1 and stored in a refrigerator at 4 °C away from light. The prepared AuNPs-PD-L1 was characterized by spectrometer.

### 3.3. Optimization of Experimental Conditions

In order to improve the sensitivity of the established immunochromatographic strips, it was essential to optimize different experimental conditions such as dosages of K_2_CO_3_ and PD-L1 in the preparation of AuNPs-PD-L1, PD-1 protein dilution times, dosage of AuNPs-PD-L1, loading buffer, and the ratio of HAuCl_4_ and NH_2_OH·HCl. The selections of the optimal experimental conditions were based on the T-line color as well as the peak area of the test strip reader.

Initially, in the preparation of AuNPs-PD-L1, the dosages of K_2_CO_3_ (0.2 mol/L) and PD-L1 would affect the coupling effect of AuNPs and PD-L1. Therefore, the dosages of K_2_CO_3_ and PD-L1 were optimized in this experiment. Different dosages of K_2_CO_3_ (1 μL, 2 μL, 3 μL, 4 μL, and 5 μL) and PD-L1 (0.5 μL, 1 μL, 1.5 μL, 2 μL, and 2.5 μL) were added, respectively, and the remaining experimental steps are referred to in Section 2.2. The color changes of each tube were observed; when the AuNPs were wine red without change, the dosages of K_2_CO_3_ and PD-L1 added to the tube were optimal. Then, PD-1 protein (1.66 mg/mL) was diluted at different times (10, 20, 30, 40, and 50) with buffer, and the final concentration of PD-1 protein was 0.166 mg/mL, 0.083 mg/mL, 0.055 mg/mL, 0.042 mg/mL, and 0.033 mg/mL. Immunochromatographic test strips with different T-line concentrations were prepared. In addition, different dosages of AuNPs-PD-L1 (5 μL, 10 μL, 15 μL, 20 μL, 25 μL, and 30 μL) were mixed with Tween-20 and PD-1 monoclonal antibody, respectively. The mixed solutions were added to the immunochromatographic test strips. Further, ultra-pure water, PBST, PBS, and PB were chosen as different buffers to dilute the PD-1 antibody. Tween-20, AuNPs-PD-L1, and PD-1 monoclonal antibody were mixed with different buffers and added to the immunochromatographic test strips. Furthermore, the optimal ratio of HAuCl_4_ and NH_2_OH·HCl was critical in the experiment, which might affect whether the experimental results could be read smoothly. HAuCl_4_ and NH_2_OH·HCl were mixed at the ratio of 1:5, 1:10, and 1:15. A certain dosage of AuNPs-PD-L1, 100 μL of buffer, and Tween-20 were mixed and added to the immunochromatographic test strips. Then, the enhancement solution was added to the sample pad after 5 min. The results of the test strips were observed, and the optimal experimental conditions were selected.

### 3.4. Preparation of Immunochromatographic Strips

The strips were made of sample pads, nitrocellulose membranes, absorbent pads, and PVC plywood. The nitrocellulose membranes, sample pad, and absorbent pad were assembled on the PVC plywood in turn, overlapping each other by about 2 mm. PD-1 protein was allocated to nitrocellulose membrane at a rate of 0.5 μL/cm to prepare the test area of the immunochromatographic strip, forming the T-line, and the strips were dried for 8 h at 37 °C in the oven. The strips were then cut into 4 mm wide pieces and kept in a dry environment. The assembly diagram of the immunochromatographic strip is shown in Figure S2.

### 3.5. Establishment of the AuNP Enlargement Immunochromatographic Methods

The principle of the AuNPs enlargement immunochromatographic methods is as follows: without AuNPs, NH_2_OH·HCl has a weak reducibility to HAuCl_4_, thus slowly generating AuNPs. On the contrary, in the presence of AuNPs, the reduction process between NH_2_OH·HCl and HAuCl_4_ can be catalyzed by AuNPs, thereby rapidly generating new AuNPs. These new AuNPs will rapidly deposit and cover the surface of the initial AuNPs to form larger AuNPs, which will significantly amplify the signal at the T-line of the strip [27].

**Figure 4:**
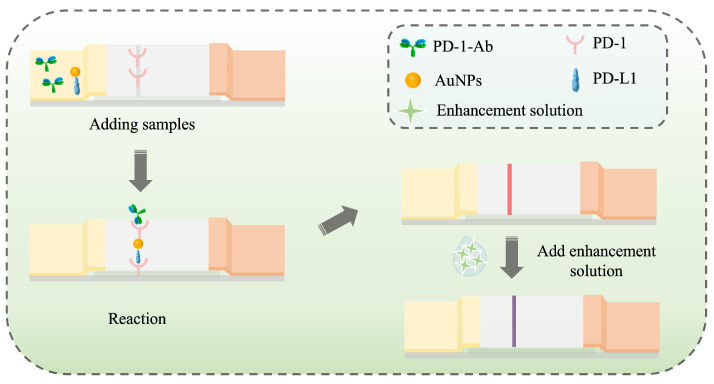
Flow chart of immunochromatographic test strips for detecting the PD-1 monoclonal antibody concentration.

Figure 4 depicts the process of detecting the concentration of PD-1 monoclonal antibody on a strip. The mixed solution of PD-1 monoclonal antibody, AuNPs-PD-L1, and Tween-20 was dripped onto the sample pad, and the mixed solution flowed forward due to driving capillary action. According to the specific antigen and antibody recognition, the PD-1 protein fixed on the nitrocellulose membrane was captured, resulting in the accumulation of AuNPs on the surface of the area, and the excess AuNPs continued to flow until being absorbed [28]. The color of the T-line was red at this time. Then, the enhancement solution (a solution of NH_2_OH·HCl and HAuCl_4_ mixed in a certain ratio) was dripped onto the sample pad. The newly generated AuNPs were accumulated onto the T-line to form the larger diameter of AuNPs, significantly enhancing the signal on the T-line. The color of the T-line became purple at this time.

### 3.6. Test Strip Reader

A portable test strip reader was developed, which could match the immunochromatographic test strip. The reader used the photoelectric sensing system to detect the reflected light on the test samples using a light-emitting diode, and the photoelectric sensor converted the light signal into an electrical signal and output the detection data after processing. By using the reader to distinguish the color change of the test strip, the subjective error caused by human interpretation could be avoided, and the test results could be obtained quickly, with high accuracy and repeatability. The concentration of the PD-1 monoclonal antibody was detected by measuring the reflected light intensity to reflect the color of the strip. Using the peak area method, the vertical coordinate was the peak area, and the horizontal coordinate was the experimental parameters of the PD-1 monoclonal antibody, plotting the experimental results.

### 3.7. Analytical Performance of Immunochromatographic Test Strips

While under the selected optimal experimental conditions, the detection limit, the specificity, the stability, the repeatability, the accuracy, and the applicability of the immunochromatographic strips for PD-1 monoclonal antibody were assessed to indicate analytical performance of immunochromatographic test strips.

Firstly, 10 μL of Tween-20, 20 μL of AuNPs-PD-L1, and 90 μL of PD-1 antibodies were mixed and added to the immunochromatographic test strips. Then, the strips were left for 10 min, and the color changes of the T-line were observed. Then, 1% HAuCl_4_ and NH_2_OH·HCl were mixed at a certain ratio as enhancement solution. Next, 100 μL of enhancement solution was added to the sample pad, and the color changes of T-line were observed after being left for 5 min. According to the principle of competitive immunochromatography, when the concentration of PD-1 monoclonal antibody was 0 mg/mL, the color of the T-line should be the darkest, and with the increase in the concentration of PD-1 monoclonal antibody, the color of the T-line gradually became lighter until disappearing. At this time, the concentration of PD-1 monoclonal antibody was the detection limit of the immunochromatography strips.

In addition, the prepared immunochromatographic strips were used to detect PD-1 monoclonal antibody, BSA, OVA, and other samples. PD-1 monoclonal antibody, BSA, and OVA were added, respectively. Their concentrations were the same, 0.5 mg/mL. If the color of the T-line became lighter, it indicated that there was a reaction. The color change of the T-line was observed, and the specificity of the immunochromatographic strip was evaluated.

Further, the prepared immunochromatographic strips were placed in a plastic bag equipped with desiccants and placed at room temperature. The immunochromatographic strips were detected at 1, 2, 3, 4, 5, 6, and 12 months after storage. The stability of the immunochromatographic strips was evaluated according to the color change of T-line.

Moreover, four different batches of immunochromatographic strips, A, B, C, and D, were prepared. The detection results were compared, and the repeatability of the immunochromatographic strip was evaluated according to the color change of the T-line.

Moreover, different concentrations of PD-1 monoclonal antibody standard were added to the PBS buffer and human serum. The recovery rates and relative standard deviations were calculated by both ICTS method and ELISA method to evaluate the accuracy.

Lastly, two kinds of real samples were detected by both ICTS method and ELISA method to test the applicability of the prepared immunochromatographic strips.

## 4. Conclusions

In this work, a rapid, simple, and sensitive immunochromatographic strip (ICTS) was developed for visual and instrumental detection of the concentration of the PD-1 monoclonal antibody. This study also features the establishment of AuNPs enlargement immunochromatographic methods. Further, the establishment of the AuNPs enlargement methods improved the sensitivity of PD-1 monoclonal antibody detection based on traditional AuNPs. After optimization, HAuCl_4_ and NH_2_OH·HCl were added to the sample pad in the ratio of 1:10. Under the optimal conditions, the LOD was 1.58 ng/mL with a triple signal-to-noise ratio. The entire testing process took only 10 min to obtain results, which was significantly shorter than other testing methods. The visual quantitative detection standard curve of PD-1 monoclonal antibody concentration was constructed by using the test strip reader. The constructed immunochromatographic strip had good stability, repeatability, and specificity and can rapidly, accurately, and efficiently detect the concentration of the PD-1 monoclonal antibody in real samples.

## Figures and Tables

**Table 1:** Accuracy and precision of immunochromatographic test strips.

	Addition Concentration (mg/mL)	Measurement Value (mg/mL)	Average Value (mg/mL)	Recovery Rate (%)	Relative Standard Deviation (%)
PBS buffer	10.0	10.63 10.77 11.67 10.57 9.99 10.81	10.74	107.40	5.06
	1.0	0.99 1.11 0.91 0.99 1.01 1.13	1.02	102.33	8.07
	0.1	0.09 0.09 0.10 0.09 0.09 0.11	0.10	95.00	8.81
human serum	10.0	9.57 9.73 10.20 10.32 9.82 10.21	9.93	99.28	3.10
	1.0	1.09 1.06 0.97 0.99 0.89 0.86	0.98	97.67	9.29
	0.10	0.09 0.09 0.1 0.11 0.1 0.09	0.01	96.67	8.45

**Table 2:** Application of immunochromatographic test strips to real samples (n = 3).

Samples	Acutal Concentration (mg/mL)	ICTS Method		ELISA Method	
		Measurement Value (mg/mL)	Relative Standard Deviation (%)	Measurement Value (mg/mL)	Relative Standard Deviation (%)
Sample 1	5.0	4.82	2.04	4.93	1.87
Sample 2	10.0	9.82	1.43	9.68	1.49

## Data Availability

The datasets generated for this study are available on request to the corresponding author.

## Supplementary Materials

The following supporting information can be downloaded at: https://www.mdpi.com/article/10.3390/molecules29133046/s1, Figure S1: Assembly diagram of immunochromatographic test strips; Figure S2: Optimization of experimental conditions; Table S1: Comparison of the methods for the detection of PD-1 monoclonal antibody drugs [16,17,18]; Table S2: Accuracy and precision of ELISA methods.
