# Supplementary material for: An Immunochromatographic Test Strip for Rapid Quantitative Control of Monoclonal Antibodies against Programmed Cell Death Protein 1
Source: Molecules. 2024 Jun 27;29(13):3046. doi: 10.3390/molecules29133046 (PMC11243010; doi:10.3390/molecules29133046)
Supplement: Supplementary file 1 [file molecules-29-03046-s001.zip › molecules-3022266-supplementary.pdf]

Supplementary Information

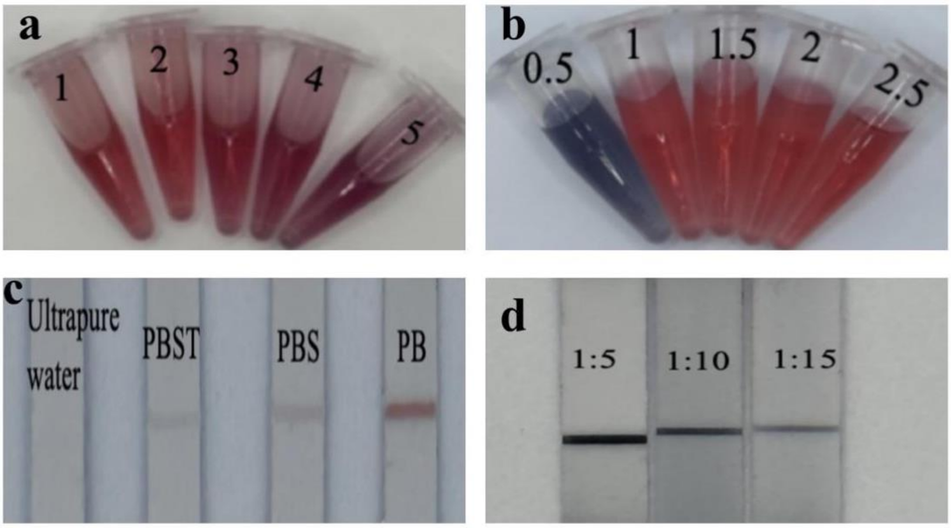

Figure S1. Optimization of experimental conditions. Optimization of the dosages of (**a**) K_2CO_3 and (**b**) PD-L1 in the preparation of AuNPs-PD-L1, (**c**) Optimization of the loading buffer, (**d**) Optimization of the ratio of $HAuCl_4$ and $NH_2OH{\cdot}HCl$.

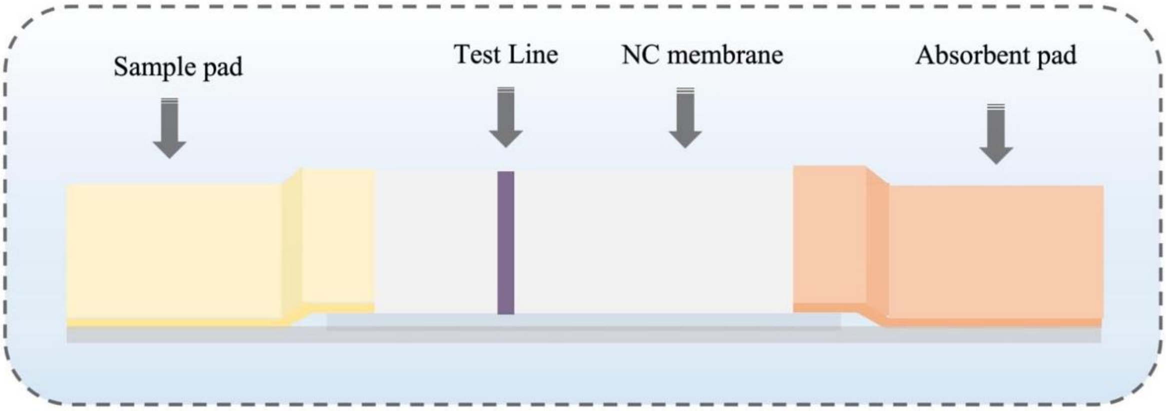

Figure S2. Assembly diagram of immunochromatographic test strips.

Table S1. Comparison of the methods for the detection of PD-1 monoclonal antibody drugs.

Detection method	LOD	Samples	References
ELISA	5 g/mL	Nivolumab	[1]
ELISA	2 ng/mL	Pembrolizumab	[2]
LC-MS/MS	0.977 g/mL	Nivolumab	[3]
ICTS	1.58 ng/mL	Pembrolizumab	This assay

Table S2. Accuracy and precision of ELISA methods.

	Addition concentration (mg/mL)	Average value ± SD (mg/mL)	Recovery rate（%)	Relative standard deviation (%)
PBS buffer	10	10.12 ± 0.21	101.15	2.09
	1	0.99 ± 0.10	99.33	9.84
	0.1	0.1 ± 0.01	99.67	8.45
Human serum	10	9.97 ± 0.14	99.68	1.37
	1	1.01 ± 0.10	101.33	9.50
	0.1	0.10 ± 0.01	98.33	7.65

References

1. Puszkiel, A.; Noé, G.; Boudou-Rouquette, P.; Cossec, C.L.; Arrondeau, J.; Giraud, J.S.; Thomas-Schoemann, A.; Alexandre, J.; Vidal, M.; Goldwasser, F.; et al. Development and validation of an ELISA method for the quantification of nivolumab in plasma from non-small-cell lung cancer patients. Journal of pharmaceutical and biomedical analysis 2017, 139, 30-36.
2. Pluim, D.; Ros, W.; van Bussel, M.T.J.; Brandsma, D.; Beijnen, J.H.; Schellens, J.H.M. Enzyme linked immunosorbent assay for the quantification of nivolumab and pembrolizumab in human serum and cerebrospinal fluid. Journal of pharmaceutical and biomedical analysis 2019, 164, 128-134.
3. Iwamoto, N.; Shimada, T.; Terakado, H.; Hamada, A. Validated LC-MS/MS analysis of immune checkpoint inhibitor Nivolumab in human plasma using a Fab peptide-selective quantitation method: nano-surface and molecular-orientation limited (nSMOL) proteolysis. Journal of chromatography. B, Analytical technologies in the biomedical and life sciences 2016, 1023-1024, 9-16.
